# Everolimus-related unilateral abdominal lymphedema in a renal cancer patient: A case report

**DOI:** 10.1097/MD.0000000000022634

**Published:** 2020-10-16

**Authors:** Jana Halamkova, Tomas Kazda, Dagmar Adamkova-Krakorova, Sylva Rybnickova, Igor Kiss, Regina Demlova

**Affiliations:** a Department of Cancer Comprehensive Care, Masaryk Memorial Cancer Institute, Faculty of Medicine Masaryk University; b Department of Medical Ethics, Faculty of Medicine, Masaryk University; c Department of Radiation Oncology, Masaryk Memorial Cancer Institute, Faculty of Medicine Masaryk University; d Department of Cancer Comprehensive Care; e Department of Radiology, Masaryk Memorial Cancer Institute; f Clinical Trial Unit, Masaryk Memorial Cancer Institute; g Department of Pharmacology, Faculty of Medicine, Masaryk University, Brno, Czech Republic.

**Keywords:** abdominal lymphedema, case report, everolimus-related adverse event, mammalian target of rapamycin inhibitors, renal cancer, unilateral lymphedema

## Abstract

**Rationale:**

Unilateral manifestation of lymphedema during everolimus therapy has been described only rarely, mostly in transplant recipients.

**Patient concerns:**

We report the first case of a patient who developed unilateral abdominal lymphedema, during a short period of everolimus treatment for renal cancer.

**Diagnosis:**

The abdominal asymmetry occurred only on the right side of the abdomen, neither ultrasound nor CT scan detected ascites but showed enlargement of the abdominal wall. The Naranjo Adverse Drug Reaction Probability scale was evaluated, in this case, a score of 6 indicated a probable adverse reaction to everolimus.

**Interventions:**

Discontinuation of everolimus therapy led to immediate alleviation and reduction of the lymphedema, with worsening once again after initiating retreatment with everolimus at a reduced dose.

Outcomes: The patient's lymphedema recovered after discontinuation of everolimus.

**Lessons:**

This rare case demonstrates the importance of the selection of mammalian target of rapamycin inhibitors using caution, especially for patients with a high risk of developing lymphedema.

## 1 Introduction

Everolimus is a rapamycin ester analog, an orally bioavailable inhibitor of the mammalian target of rapamycin (mTOR), which has proven benefits in the areas of transplantation and oncology. While everolimus is a well-tolerated drug, related adverse events include nausea, vomiting, mucositis, diarrhea, fatigue, hyperlipidemia, hyperglycemia, hypertension, oedema and an increased incidence of lymphoceles. A more serious but less frequent side effect is non-infectious pneumonitis, which is observed in the group of rapamycin analogs.

The mTOR is a component of an intracellular signaling pathway regulating cell growth and proliferation, metabolism, and angiogenesis. It is a downstream effector of the phosphatidylinositol-3-kinase/protein kinase B/mTOR pathway, which is an intracellular signaling pathway important in regulating the cell cycle. Abnormal functioning of the mTOR pathway may contribute to the pathogenesis of some solid tumors, especially renal cell carcinoma (RCC).^[1]^ The mTOR is the molecular target for small molecule inhibitors (i.e., temsirolimus and everolimus), which have demonstrated significant clinical activity in patients with advanced RCC. Preclinical and clinical studies show that everolimus inhibits the proliferation of a variety of human solid tumors and reduces the expression of vascular endothelial growth factor (VEGF).^[2,3]^ Rapamycin also significantly inhibits lymphatic endothelial cell (LEC) proliferation and migration.^[4]^

Bilateral oedemas are a known complication, but their unilateral manifestation has been described only rarely, mostly in transplant recipients.^[5,6]^ A less-common type of adverse event as a result of mTOR inhibitor therapy is lymphedema. The spontaneous formation of lymphedema has been seen with sirolimus-based immunosuppression in renal transplant recipients^[7–9]^ but unilateral lymphedema is an extremely rare complication of everolimus treatment, and up to now has only been documented in 4 cases of transplant recipient patients receiving long term therapy of everolimus at lower doses than the maximum 2 mg/d.^[5,7,10]^ Here we report the first case of a patient who developed unilateral lymphedema of the abdominal wall during a short period of everolimus treatment for renal cancer.

## 2 Case report

**Figure 1:**
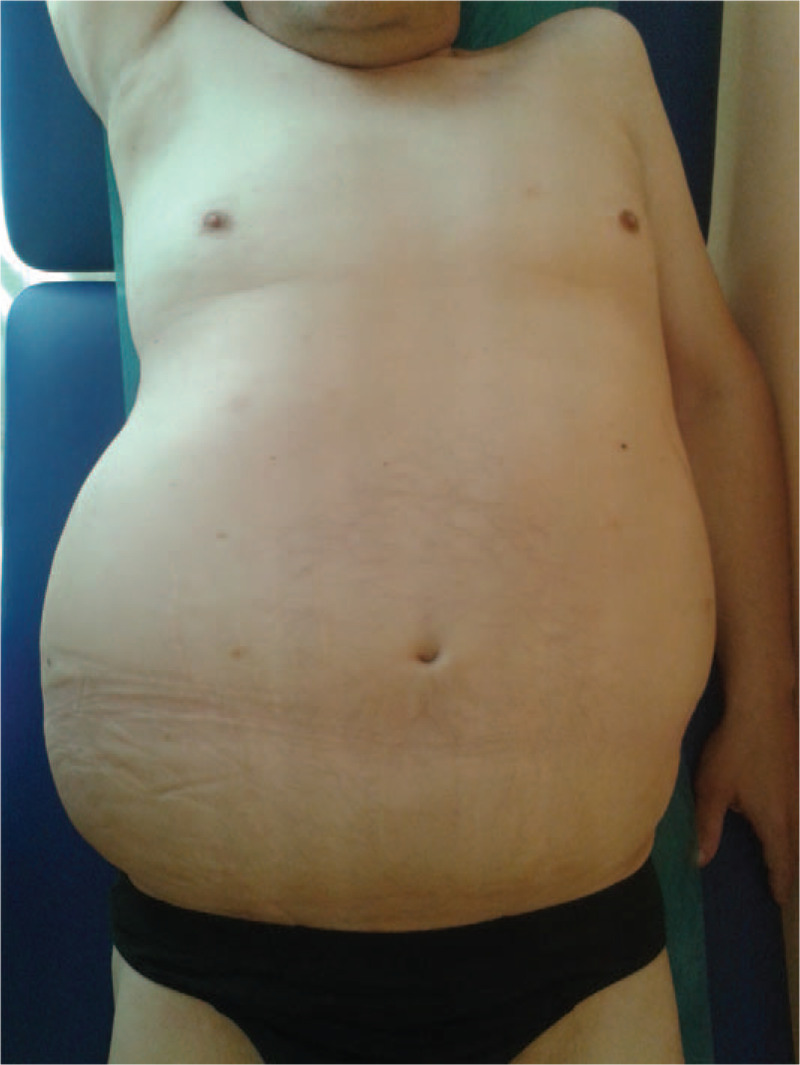
Unilateral lymphedema of the abdominal wall - asymmetry of the abdomen.

**Figure 2:**
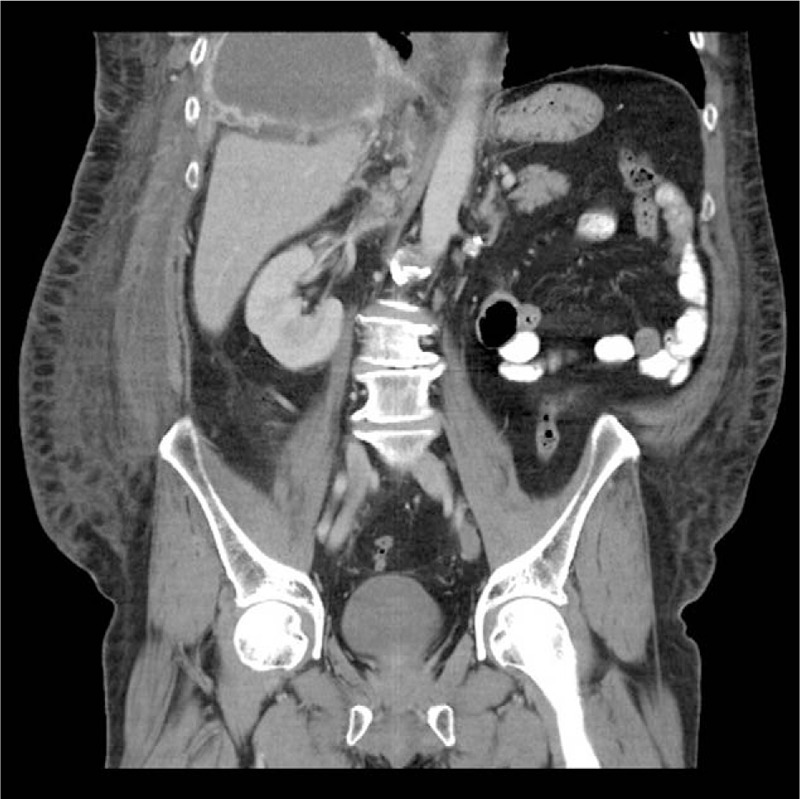
Sagittal contrast-enhanced computed tomography of lymphedema on the right side of the abdomen.

**Figure 3:**
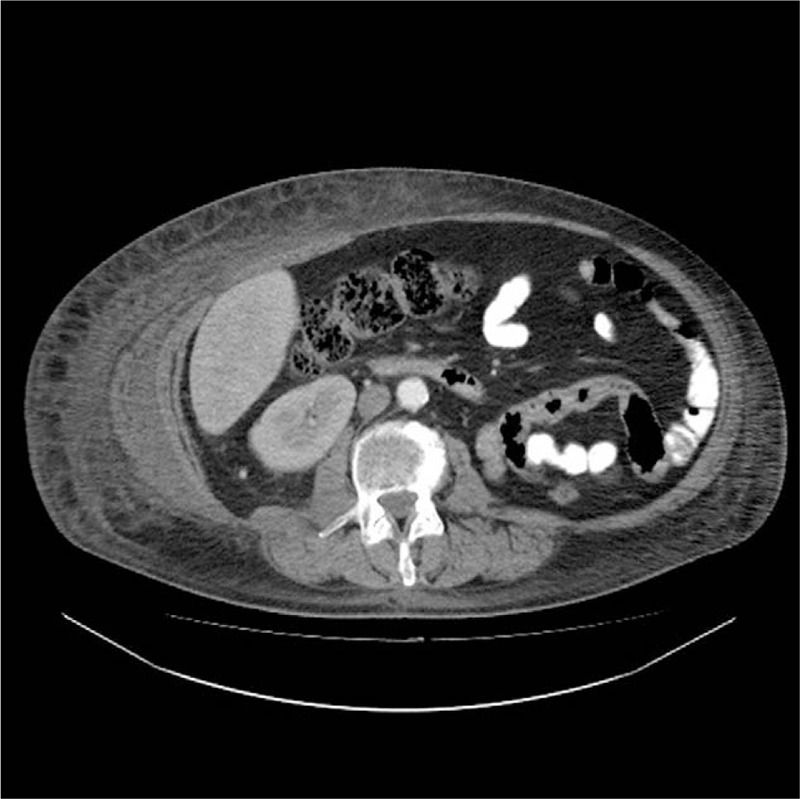
Axial contrast-enhanced computed tomography of lymphedema on the right side of the abdomen.

A 49-year old male patient, without any previous history of serious medical disorders, with a RCC (Grawitz tumor), was diagnosed as being in the metastatic stage in March 2014, following left nephrectomy. Treatment by the tyrosine kinase inhibitor sunitinib (50 mg/d – 4 weeks on/ 2 weeks off) for bone, pulmonary and pleural metastases was initiated. After 9 months of the treatment, the patient developed solitary cerebral metastases, confirmed by CT scan. Therapy by neurosurgery and radiotherapy were subsequently performed. In order to contain the progression of the disease, systemic therapy of everolimus 10 mg/d was commenced in January 2015. The renal cancer remained stable but in May 2015 a unilateral volume of the right part of the abdominal wall began increasing, in the form of lymphedema, emanating from the right axilla to the right inguina (Fig. 1). The patient indicated pain, oedema and uncomfortable stiffness of the abdominal wall. The abdominal asymmetry occurred only on the right side of the abdomen, neither ultrasound nor CT scan detected ascites but showed enlargement of the abdominal wall (Figs. 2 and 3). Oedema was not detected in either the lower or upper extremities and the lymphedema progressed considerably over several days. CT scan failed to reveal the cause of the unilateral abdominal oedema unexplained. A therapy of diuretics was initiated, for the symptomatic reduction of the volume of the abdominal wall. This treatment (furosemide 40 mg/d) and higher doses of corticosteroids (dexamethasone 24 mg/d) had no effect, however discontinuation of everolimus therapy led to immediate alleviation and reduction of the oedema. Everolimus was not replaced by another medicine during the discontinuation, but worsening of the oedema after initiating retreatment with everolimus at a reduced dose 5 mg/d was observed. The Naranjo Adverse Drug Reaction Probability Scale^[11]^ was evaluated, in this case, a score of 6 indicated a probable adverse reaction to everolimus. The lymphedema was significant enough to affect the level of comfort and had significantly reduced the quality of life for the patient.

## 3 Discussion

The mammalian VEGF family is comprised of 5 members: VEGF A, VEGF B, VEGF C, VEGF D, and placenta growth factor. It has been described that a number of proteins are structurally related to VEGF encoded by parapoxviruses (VEGF E) and in snake venom (VEGF F). The VEGF ligands can bind to 3 receptor type tyrosine kinases: vascular endothelial growth factor receptor (VEGFR)l/FLT1, VEGFR2/kinase insert domain receptor, and VEGFR3/Fms-related tyrosine kinase 4. VEGF A, B, and placenta growth factor can bind to VEGFR1, VEGF A, and E bind to VEGFR2, while VEGF C, and D bind to VEGFR3.^[12]^

Rapamycin and its derivatives (mTOR inhibitors) potently inhibit VEGF C driving proliferation and migration, respectively, of isolated human LECs in vitro. mTOR inhibition impairs downstream signalling of VEGF A as well as VEGF C by binding mTOR to p70S6 kinase in LECs, mTOR-mediated phosphorylation of p70S6 kinase is crucial for impairing both the VEGF A- and VEGF C-mediated proliferation of LECs.^[4]^

The lymphatic endothelium expresses VEGFR3, which is activated after binding to VEGF C, and VEGF D and plays an important role in lymphangiogenesis. The gene that encodes VEGFR3 is defective in most families with congenital hereditary lymphedema^[13]^ and impaired lymphangiogenesis and lymphedema is observed in soluble VEGFR3 (VEGF C/VEGF D signaling inhibitor) expressing transgenic mice.^[14]^ Missense mutations of VEGFR3 prevent normal lymphatic growth in humans.^[4]^ In rapamycin-treated animals, the anti lymphangiogenic effect during tissue regeneration occurs with prolonged lymphedema and lymphatic neovascularization is markedly inhibited, thus emphasizing the clinical relevance of this effect of mTOR inhibition.^[4]^ mTOR inhibition potently decreases regenerative and neoplastic lymphangiogenesis. Bilateral mTOR-related oedemas are usually controlled with low doses of furosemide accompanied by reducing the immunosuppressant, but not in lymphedema.^[15]^

mTOR inhibitors rarely cause lymphedema by inhibiting different subtypes of VEGFs, which results in impaired lymphangiogenesis. While there is a small number of reports about everolimus-related unilateral lymphedema, this case represents the first everolimus-related unilateral lymphedema in a cancer patient.^[5–7]^ It is important to note that in the literature describing mTOR-related lymphedema, it occurs mainly on the postsurgical side,^[16]^ whereas this is not our case, as the nephrectomy was on the left side.

It could be that mTOR inhibitors interfere with other, as yet unknown, pathways that operate to induce lymphangiogenesis, and maintain lymphatic integrity. In patients with pre-existing lymphatic weakness, everolimus, through its action on this pathway, may inhibit lymphangiogenesis to a degree that manifests as clinical lymphedema. Everolimus is currently approved for oncology patients in the therapy of renal cancer, neuroendocrine tumours and breast cancer (concomitantly with aromatase inhibitors). Especially for patients with breast cancer, where lymphedema of the upper extremities is a common adverse event of breast surgery and radiotherapy, those patients with a predisposition for the development of this unexpected side effect should be carefully screened. Finally, the life-threatening complications of the treatment, such as angioedema, should be considered.^[17]^

In conclusion, we observed a very rare side effect of treatment with everolimus, unilateral abdominal lymphedema in a cancer patient, due to the mTOR pathway being interfered with. Inhibitors of the mTOR pathway must be prescribed with caution, especially to patients at high risk of developing lymphedemas.

## Author contributions

All authors read and approved the final manuscript.

**Conceptualization:** Jana Halamkova.

**Methodology:** Jana Halamkova, Dagmar Adamkova-Krakorova, Sylva Rybnickova, Tomas Kazda.

**Resources:** Jana Halamkova, Regina Demlova

**Writing – original draft:** Jana Halamkova

**Writing – review & editing:** Jana Halamkova, Igor Kiss
